# Lower visual processing speed relates to greater subjective cognitive complaints in community-dwelling healthy older adults

**DOI:** 10.3389/fpsyt.2023.1063151

**Published:** 2023-03-21

**Authors:** Daniela Marrero-Polegre, Kathrin Finke, Naomi Roaschio, Marleen Haupt, Cristian Reyes-Moreno, Adriana L. Ruiz-Rizzo

**Affiliations:** ^1^General and Experimental Psychology Unit, Department of Psychology, LMU Munich, Munich, Germany; ^2^Department of Neurology, Jena University Hospital, Jena, Germany; ^3^Department of Education and Psychology, Freie Universität Berlin, Berlin, Germany; ^4^Experimental Psychology Lab, Department of Psychology, Carl von Ossietzky University of Oldenburg, Oldenburg, Germany

**Keywords:** aging, memory complaints, subjective function, subjective cognitive decline, visual attention, visual processing speed

## Abstract

**Introduction:**

Subjective cognitive complaints in older age may reflect subtle objective impairments in basic cognitive functions that might foreshadow broader cognitive problems. Such cognitive functions, however, are not captured by standard neuropsychological testing. Visual processing speed is a basic visual attention function that underlies the performance of cognitive tasks relying on visual stimuli. Here, we test the hypothesis that lower visual processing speed correlates with greater subjective cognitive complaints in healthy older adults from the community.

**Methods:**

To do so, we assessed a sample of 30 healthy, cognitively normal older adults (73.07 ± 7.73 years old; range: 60–82; 15 females) with respect to individual subjective cognitive complaints and visual processing speed. We quantified the degree of subjective cognitive complaints with two widely-used questionnaires: the Memory Functioning Questionnaire and the Everyday Cognition. We used verbal report tasks and the theory of visual attention to estimate a visual processing speed parameter independently from motor speed and other visual attention parameters, i.e., visual threshold, visual short-term memory storage capacity, top-down control, and spatial weighting.

**Results:**

We found that lower visual processing speed correlated with greater subjective complaints and that this relationship was not explained by age, education, or depressive symptoms. The association with subjective cognitive complaints was specific to visual processing speed, as it was not observed for other visual attention parameters.

**Discussion:**

These results indicate that subjective cognitive complaints reflect a reduction in visual processing speed in healthy older adults. Together, our results suggest that the combined assessment of subjective cognitive complaints and visual processing speed has the potential to identify individuals at risk for cognitive impairment before the standard tests show any abnormal results.

## 1. Introduction

Visual processing speed is a basic visual attention function that influences global cognition (1, 2) and decreases in typical aging (3–6) and in individuals at risk for dementia (7–9). Many everyday activities involve dealing with multiple visual stimuli, thereby requiring efficient visual processing. Accordingly, reduced visual processing speed can negatively impact older adults’ independence in daily living (10, 11). Reduced visual processing speed has been shown to explain difficulties in simultaneous object perception in patients with amnestic mild cognitive impairment and dementia (9, 12). Reduced visual processing speed could also explain the reduced benefit from perceptual detail in visual scenes during memory recognition in healthy older adults (13), given that one aspect of perceptual detail in visual scenes is defined by the number of objects contained in them (14). Thus, reduced visual processing speed might foreshadow broader cognitive impairments. *Subjective* cognitive complaints, commonly observed in older adults, have been linked with reduced *objective* cognitive performance (15), i.e., steeper rates of decline in verbal memory (16) and lower performance in attention, executive functions, and language tasks (17, 18). However, subjective cognitive complaints can indicate cognitive impairment *before* it becomes obvious on neuropsychological tests (19–22). Thus, subjective cognitive complaints might reflect subtle impairments in everyday activities that might result from slowed visual processing. In the present study, we investigate visual processing speed as a potential neurocognitive correlate of subjective cognitive complaints in community-dwelling healthy older adults.

Accurately measuring visual processing speed requires sensitive and specific tools (23, 24). Extant neuropsychological measures (e.g., trail-making test A, digit-symbol substitution, pattern comparison, among others) are useful tools sensitive to aging effects (25, 26). However, their specificity might be reduced due to their reliance on fast motor responses or other cognitive processes, all of which become impaired with aging. The theory of visual attention (TVA) (27) is a mathematical model that allows estimating visual processing speed independently from visual short-term memory (VSTM) storage capacity, top-down attentional control, spatial bias in attention, and visual perceptual threshold (27) based on performance in two psychophysical verbal report tasks that do not rely on motor response speed. In more detail, in TVA, the processing rate of an object in the visual field (e.g., a letter) is defined by its probability of being encoded in VSTM, i.e., selected and categorized (27). An object’s processing rate depends on the bias toward a perceptual category, which is determined both by the task and an individual’s alertness level (28). The sum of the processing rate of all objects in the visual field determines a parametric measure of the visual processing capacity of an individual and is given in the number of items per second (27). In practice, this parameter can be obtained from the unspeeded verbal report of letter arrays presented under varying exposure durations, to which the TVA model is fitted. Thus, performance does not directly depend on motor speed, which is a clear advantage in assessing visual attention in older adults. Specific neural correlates in brain connectivity have been shown for this TVA-based visual processing speed parameter in healthy older adults (6, 29). More specifically, the cingulo-opercular (e.g., insula and anterior cingulate cortex) and the right frontoparietal (e.g., dorsal frontal and parietal cortices) network have been identified as relevant for visual processing speed. Therefore, the TVA model provides a well-defined, adequate measure of visual processing speed in older adults.

In the present study, we aimed to determine the association between visual processing speed and subjective cognitive complaints in community-dwelling healthy older adults. To do so, in a sample of healthy, cognitively normal older adults recruited from the community, we obtained the visual processing speed parameter based on the TVA model. We used a task paradigm in which some of the trials were preceded by an alerting tone cue, following recent studies on mild cognitive impairment and visual processing speed (8). While we included both cued and uncued trials, cueing effects were not analyzed for the present study (see section “2.4.1 Measurement”). We also computed an overall subjective cognitive complaint score based on two structured questionnaires. We hypothesized that lower visual processing speed would be associated with greater subjective cognitive complaints. We based our hypothesis on the well-known slowing of visual processing and the presence of subjective cognitive complaints in healthy aging individuals and the influence of visual processing speed on global cognitive function and older adults’ independence as outlined above. Moreover, greater cognitive complaints have been associated with increased activity in insular, lingual, and cerebellar areas during memory tasks (16). The overlap between some of these brain regions with those associated with visual processing speed (6) might also support the link between visual processing speed and subjective cognitive complaints (16). To test the specificity of this link, we tested control associations of the other TVA parameters (i.e., VSTM storage capacity, top-down attentional control, and spatial weighting) with subjective cognitive complaints. We additionally measured depressive symptoms and personality traits such as neuroticism and conscientiousness because these are relevant factors that might give rise to subjective cognitive complaints (30) or subjective cognitive decline (i.e., subjective cognitive complaints *and* concerns about them in the absence of objective impairment in standardized tests) (21, 22).

## 2. Materials and methods

### 2.1. Participants

Thirty-two older adults (mean age: 72.53 ± 7.78 years; range: 57–82; 16 females) were invited to participate. Potential volunteers were recruited from lists including participants of previous studies at LMU Munich (Munich, Germany) who had agreed on being contacted again as well as from word-of-mouth. Selection criteria were 55 years of age or older; being a German native speaker; demonstrating normal performance in standard neuropsychological tests (see “Objective cognitive performance”); no psychiatric or neurological disorders, including mild cognitive impairment or dementia; no history of head trauma; and normal or corrected-to-normal vision and hearing, as indicated by self-report. Although the presence of a subjective feeling of memory worsening or cognitive decline was neither advertised for participant recruitment nor used as a participant selection criterion, three questions about that feeling were asked in the demographic questionnaire (see “Subjective cognitive complaints”). Two participants were excluded due to chronic fatigue syndrome reported in the demographic questionnaire during the first session (*n* = 1) and low performance in neuropsychological testing (i.e., < 3rd percentile in both verbal and non-verbal memory tests; *n* = 1). Thus, the final sample consisted of 30 healthy older adults (73.07 ± 7.73 years old; range: 60–82; 15 females; mostly right-handed: Edinburgh handedness inventory 85.37 ± 25.74^1^; schooling: 11 ± 2.68 years). Four participants (13.3%) reported a family history of dementia. All study participants signed the informed consent before taking part in the study and received monetary compensation for their participation after finishing each session (i.e., the neuropsychological and the TVA session). The study was approved by the Ethics Committee of the Faculty of Psychology and Education of LMU Munich (Munich, Germany) and was conducted in accordance with the Declaration of Helsinki.

### 2.2. Objective cognitive performance

To confirm that participants exhibited no objective cognitive impairment, they were first tested with a battery of standard neuropsychological tools. We used Addenbrooke’s Cognitive Examination (ACE III) (31) to assess overall cognition, including attention, memory, verbal fluency, language, and visuospatial abilities. To test episodic verbal memory encoding and storage, we used the German version of the Verbal Learning and Memory Test (VLMT) (32). Visuomotor speed and divided attention/executive function were measured with the Trail Making Test A and B (33), respectively. We used the Rey-Osterrieth Complex Figure Test (34, 35) to assess visuoconstruction, planning, and short-term visual memory, and the Stroop Color Word Test (36, 37) for executive function. Finally, the multiple-choice vocabulary intelligence test (*Mehrfachwahl-Wortschatz Intelligenz Test*) (38) was used to quantify crystallized intelligence and required participants to identify and cross out the existing word from each of 37 five-item sets that also included pseudowords. To be included in the study, participants should not score below 1.5 standard deviations (SD) of the mean expected for an individual of similar age and education, based on published normative data for each test. Participants’ handedness was measured with the Edinburgh handedness inventory (39). We also calculated an “overall objective performance” score by scaling each test to obtain participants’ *z*-score, based on the mean and standard deviation (SD) of each test, and then averaging across all scores. The objective cognitive assessment was conducted by psychologists with ample experience in neuropsychological testing and specific training for the present study. The neuropsychological testing session always preceded the TVA-based tasks.

### 2.3. Subjective cognitive complaints

After the neuropsychological testing, the degree of subjective cognitive complaints was assessed with two structured, self-report questionnaires previously used and reported in the literature, namely: the Everyday Cognition (ECog) (40) and the Memory Functioning Questionnaire (MFQ) (41). First, the ECog was used to quantify participants’ current subjective perception of their own memory, language, visual and spatial perception, and executive functions (planning, organization, and divided attention), compared to 10 years ago. The degree of current everyday functioning was measured with 39 questions rated on a four-point scale, from 1 (“better or no change”) to 4 (“consistently much worse”). Total sum scores could thus range between 39 and 156. The first question of the ECog is a general yes/no question regarding worries about memory or other cognitive problems. The response to this question was used for descriptive purposes. Second, the MFQ was used to quantify participants’ subjective perception of their memory function through seven scales; namely: general rating, retrospective functioning, frequency of forgetting, frequency of forgetting during reading, remembering past events, seriousness of forgetting, and mnemonics usage. The MFQ included 63 questions rated on a seven-point scale, from 1 (“much worse”) to 7 (“much better”). Total sum scores could thus range between 63 and 441, where a *lower* score indicated *greater* cognitive complaints. The ‘general rating’ scale (first question) was not included in the MFQ total score but was used for descriptive purposes. In the present study, total scores in each questionnaire were converted to *z*-scores by taking each questionnaire’s sample mean and SD. Given the inverse rating in each questionnaire, total MFQ *z*-scores were multiplied by −1 so that a greater *z*-score indicated greater cognitive complaints in both questionnaires. To combine the information from both questionnaires into one single measure of subjective cognitive complaints, we then averaged across both questionnaires’ *z*-scores. Finally, for descriptive purposes and because participants were not recruited from memory clinics, we included three questions about the participants’ subjective perception of memory problems in the demographic questionnaire to explicitly ask about the awareness of those problems and the potential impact of those on daily life. These questions were (a) *“Do you have the feeling that your memory is deteriorating?”: “No,” “Yes, but it does not worry me,” “Yes, it worries me”*; (b) *“In the last year, have you seen a doctor about your memory problems?”*; and (c) part 1: *“If so, did you receive a specific diagnosis?”: “Yes,” “No”;* part 2: *“Are you taking medication or are you under medical treatment for your memory problems?”: “Yes,” “No.”* A question about the family history of dementia was also included in the demographic questionnaire, as a positive family history might influence the perception of cognitive change and/or concerns.

### 2.4. Visual processing speed and other visual attention capacity parameters

#### 2.4.1. Measurement

**Figure 1:**
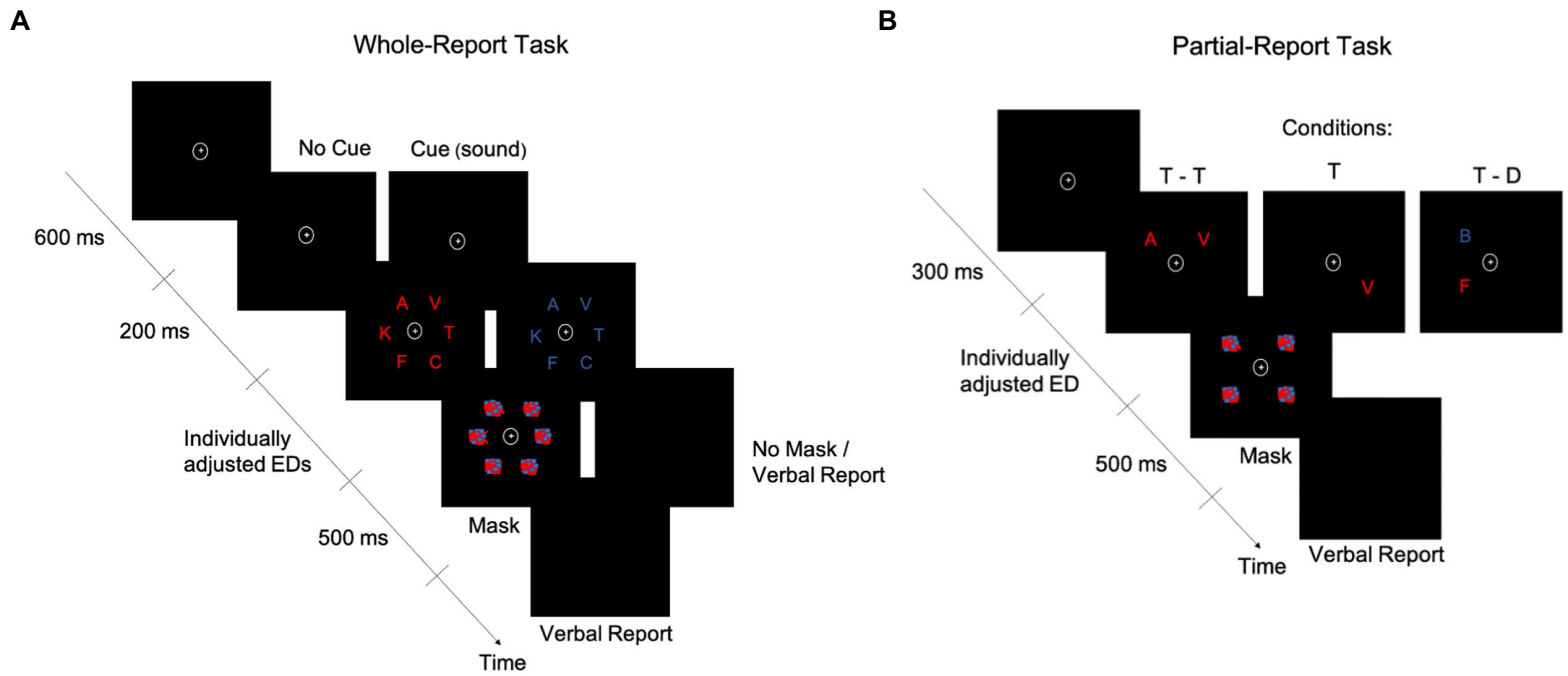
Schematics of the whole-report and partial-report tasks. A whole-report **(A)** and a partial-report **(B)** task trial are represented over time. D, distractor; ED, exposure duration; T, target.

To assess the visual processing speed parameter, we used the TVA-based psychophysical, whole-report task outlined in Haupt et al. (8) (Figure 1A). To control for other basic visual attention functions such as top-down control and spatial weighting, an additional, partial-report task (Figure 1B) was also administered. Both tasks were conducted in the second session, and multiple breaks within and between tasks were ensured (e.g., by turning on the room lights). In both tasks, stimuli were blue or red capital letters (0.88° wide × 1.06° high, or 1.0 cm wide × 1.2 cm high, each), randomly chosen from the set {A, B, C, D, E, F, G, H, J, K, L, M, N, O, P, R, S, T, U, V, W, X, Z}, and shown on a black background, with colors matched by luminosity (i.e., 0.49 cd/m^2^ (17); Figure 1). Stimuli appeared on the left and right hemifield with equal probability. Stimuli were presented on a 24-inch LED monitor (BenQ XL2411Z) with 1920 × 1080 screen resolution and a 100-Hz refresh rate in a dimly lit room. Screen brightness was set to the minimum possible and contrast was set to 50% (NVIDIA^®^ color settings). Participants were seated in front of the monitor at a viewing distance of 65 cm, and a chin rest was used. The experimenter sat next to them to type in participants’ responses on the keyboard. Both whole-and partial-report tasks were presented on a PC running Psychtoolbox-3 (v. 3.0.16) for Microsoft Windows 7, under MATLAB (v. R2017b; The Mathworks, Inc.). Each task lasted between 30 and 45 min.

In the whole-report task (Figure 1A), participants had to verbally report as many letters as they could recognize from a display that briefly presented six letters in an imaginary circle (5.64° or 6.4-cm radius). Participants were asked to fixate on the point (0.79° × 0.79° or 0.9 cm × 0.9 cm) at the center of the display at all times. After a 600-ms fixation period, letters were shown in either blue or red in a single trial. Participants did four blocks of 12 trials each to practice the task. Based on these blocks, an individual minimum exposure duration (ED) was determined for each participant (see Supplementary material for details). Four additional higher ED values were obtained from a set predefined in the task, based on the individual minimum ED. The task included 336 trials, presented in four blocks of 84 trials each. Most of the trials (i.e., 240) were followed by a masking display to control the effective ED (10), with masks consisting of scrambled squares, made of blue and red blobs. The remaining 96 trials were unmasked, which was intended to allow for iconic memory buffering (42), thereby increasing the variability in effective EDs required for reliable TVA fitting (i.e., the buffering is captured by the *μ* parameter, expressed in milliseconds and estimated from the difference in accuracy between unmasked and masked displays (28)). Accordingly, there were seven effective EDs: five with masking displays and two without masking displays (one of the second lowest and one of the highest ED). To facilitate the task for participants, they verbally reported the letters and the experimenter typed them. No emphasis was placed on the speed or order of the verbal report. In 168 of the trials, a cue^2^ tone (a 500-Hz or 900-Hz^3^ sound) was presented through headphones, with approximately equal sound volume intensity across participants. Auditory cues lasted 200 ms and appeared after the fixation point and before the letters and were intended to work as an alerting signal (17). In the uncued trials, the fixation point thus lasted 200 ms longer (i.e., 800 ms). A graphic summary bar appeared at the end of each task block, indicating the accuracy level in that block of a participant’s verbal report, i.e., the percentage of correct responses out of all responses the participant gave in that block. Based on this feedback, the experimenter told the participant how to adjust their report to avoid too liberal or too conservative responses. More specifically, if the accuracy was <70%, participants were told to try and guess *less*. If the accuracy was >90%, participants were told to guess *more* (i.e., report what they recognized without needing to be completely sure about it).

#### 2.4.2. Estimation

**Figure 2:**
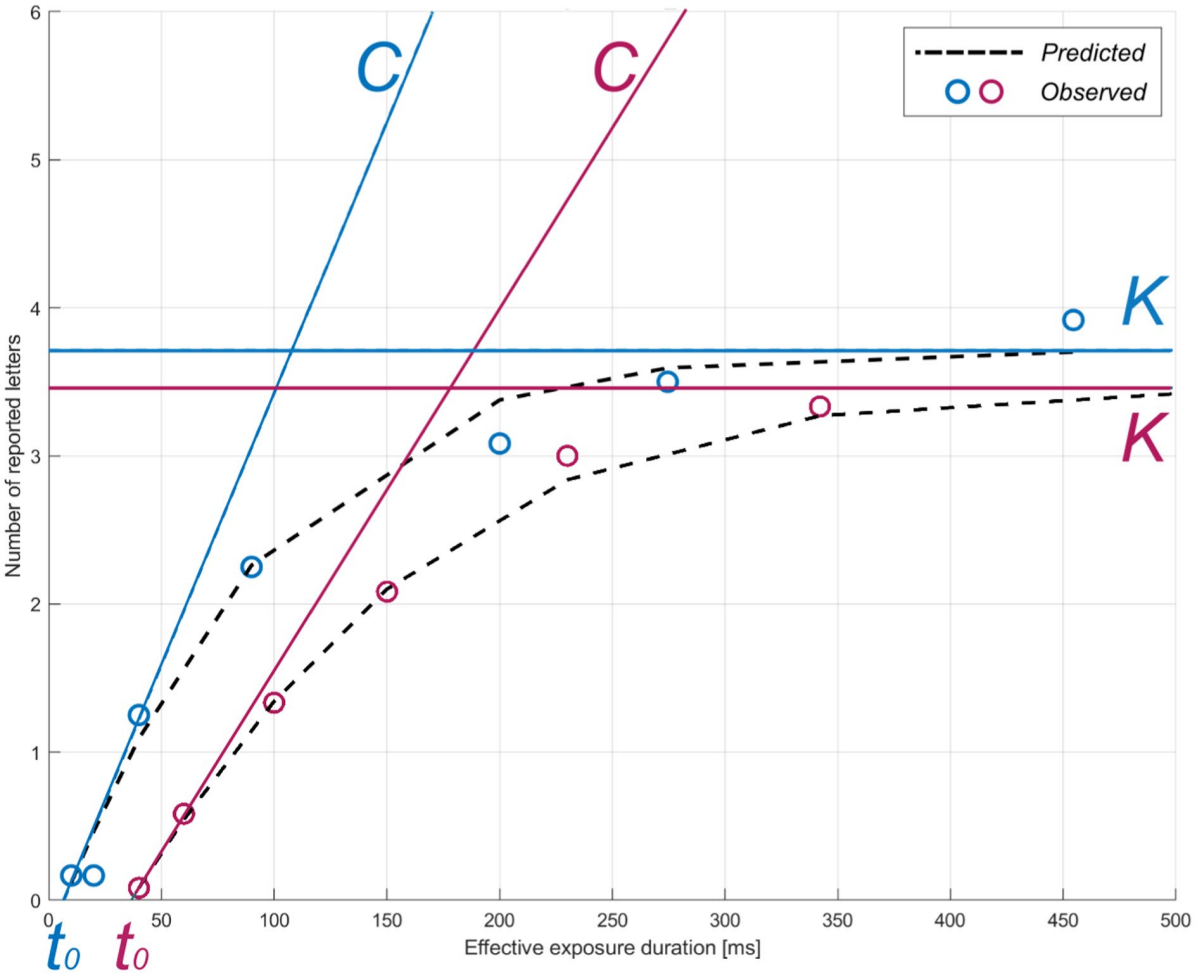
TVA parameter estimation for two representative participants. The theory of visual attention (TVA) estimation curve for accuracy as a function of exposure duration is shown for two example individuals (pink: greater subjective cognitive complaints, *z*-score = 1.09; blue: lower subjective cognitive complaints, *z*-score = −0.860) in the whole-report task. Parameters: *C* = visual processing speed; *K* = visual short-term memory storage capacity; *t_0_* = visual perceptual threshold. Circles indicate each individual’s mean observed performance across conditions. The dotted black curves indicate each individual’s predicted performance across exposure durations.

The TVA model is fitted to the verbal report in the whole-report task to obtain three parameters: visual processing speed *C* (processing rate or letters/s), VSTM storage capacity *K* (the maximum number of letters encoded in VSTM), and visual perceptual threshold *t_0_* (in ms, the threshold for conscious perception). Processing follows the exponential distribution shifted in time by *t_0_* (10). Hence, *C* is the exponential curve slope at *t_0_* and *K* is the asymptote of the curve (Figure 2). Given the task paradigm used in the present study (i.e., with and without auditory cues), two corresponding parameters for *C* were estimated. We focused on parameter *C* without auditory cues in the ensuing analyses to obtain a measure of visual processing speed that was not experimentally increased through phasic alertness (15, 17). Parameter *t0* served for the valid estimation of *C* and was thus not further considered.

### 2.5. Selection-based visual attention parameters

#### 2.5.1. Measurement

In the partial-report task (Figure 1B), participants were instructed to verbally report only the *red* letters (targets, T) while ignoring the *blue* letters (distractors, D), trying to avoid only guessing or being completely sure of their response. The experimenter typed in the participants’ responses on the keyboard. Letter displays were presented under one of three conditions: two targets (T – T), only one target (T), or a target and a distractor (T – D). To prevent a potential priming effect, the order of these conditions was pseudorandomized. Before the beginning of the actual task, an individual ED was selected for each participant. Specifically, two adjustment blocks of 24 trials each were conducted, in which participants were trained on the task. The adjustment started with an 80-ms ED and decreased by 10 ms every time the participant correctly reported T – T and increased by 10 ms if no letter was correctly reported. The final ED was decided based on the pretest accuracy (i.e., ≥ 50% for T – T trials and 70–90% for T trials). The task consisted of 288 trials in total, presented in six blocks of 48 trials each (T: 12 trials; T – T: 12 trials; T – D: 24 trials). Each target and distractor could appear in each of the four corners of an imaginary square (8.27° or 9.4-cm side) with equal frequency in a block (i.e., three times). In all trials, post-stimuli masks (scrambled squares made of blue and red blobs) were presented in all four corners, i.e., where letters were/could have been present.

#### 2.5.2. Estimation

Two parameters were estimated from the partial-report task using TVA, namely: top-down control *α* or distractibility and spatial weighting or lateralization *w_lat_*. Top-down control is computed as the attentional weight given to distractors relative to that given to targets (*w_distractors_/w_targets_*). For spatial weighting, separate attentional weights are derived for the left (*w_left_*) and the right hemifield (*w_right_*) from the accuracy of target identification in unilateral and bilateral conditions, so that it is defined as the ratio *w_left_*/(*w_right_ + w_left_*) (9). For parameter *α*, values closer to 0 indicate better top-down control, whereas values closer to 1 indicate worse top-down control. For parameter *w_lat_*, values closer to 0 indicate a leftward preference, values closer to 1 indicate a rightward preference, and values around 0.5 indicate a balanced spatial weighting.

### 2.6. Depression and personality questionnaires

We measured depressive symptoms and personality traits such as neuroticism and conscientiousness because these are relevant factors in the context of subjective cognitive complaints. Depressive symptoms were quantified through the Geriatric Depression Scale (GDS) (13), and neuroticism and conscientiousness were measured through the Big Five Inventory (BFI-10) (6).

### 2.7. Statistical analysis

Our hypothesis of an association between the parameter visual processing speed *C* and subjective cognitive complaints was tested on the performance in the task *without* auditory cues because cuing has been previously shown to influence this parameter (15, 17). However, the visual processing speed parameter obtained *with* auditory cues and other TVA parameters were also included in secondary, control analyses to confirm the specificity of our results (see Supplementary material). Given our relatively small sample, we conducted non-parametric Spearman correlation analyses to test our main hypothesis, as well as for control analyses, including partial correlations controlling for age, depressive symptoms, and education. A multiple linear regression model was used to statistically compare the associations between the four TVA parameters (i.e., *C*, *K*, *α*, and *w_lat_*: predictors or independent variables) and subjective cognitive complaints (i.e., outcome or dependent variable). The significance level was set at *ɑ* = 0.05, two-tailed. Results were Bonferroni-corrected for multiple comparisons when necessary. All data analyses were conducted in R v. 4.2.0 (43) on RStudio v. 2022.07.1 (44).

## 3. Results

### 3.1. Descriptive statistics

The sample’s performance in the neuropsychological tests and the correlation between objective performance and subjective cognitive complaints are shown in Table 1. All participants scored in the normal range, as this was an inclusion criterion. Regarding subjective cognitive complaints as asked in the demographic questionnaire, 16 participants (53.3%) reported having noticed some memory worsening but without concerns about it. Another four participants (13.3%) reported memory worsening and that they were *concerned* about it. Of the latter, only one (3.3%) had already visited a doctor (but had not received any diagnosis). According to the ECog’s first question, 40% of the participants (*n* = 12) perceived their memory as worse than before. According to the MFQ, 60% of the participants (*n* = 15) reported having “some minor memory problems” (score = 3–5/7; *n* = 5 omitted this question). The mean and SD of the total and subscale scores of ECog and MFQ are shown in Table 2. Neither age nor education significantly correlated with the ECog (both *p*-values >0.114) or MFQ scores (both *p*-values >0.115), or with overall subjective cognitive complaints (both *p*-values >0.106).

**Table 1:** Neuropsychological performance of the study sample (*n* = 30).

Test	Mean ± SD
Overall Cognition	
*z*-score across all tests	0.00 ± 0.57
Crystallized Intelligence (IQ)	126.35 ± 11.90
Addenbrooke’s Cognitive Examination III/100	94 ± 3.12
Memory	
VLMT total learning score/75	48.60 ± 8.24
VLMT delayed recall/15	10 ± 2.64
VLMT recognition/15	11.30 ± 2.56
Rey-Osterrieth Complex Figure (delayed)/36	17.27 ± 5.50
Attention	
Trail Making Test A (time in s)	39.4 ± 14.67
Visuoconstruction	
Rey-Osterrieth Complex Figure (copy)/36	32.65 ± 2.95
Executive Function	
Trail Making Test B (time in s)	94.03 ± 37.39
Stroop Test Interference (time in s)	85.53 ± 18.04
Behavioral questionnaires	
Geriatric Depression Scale/15	1.57 ± 1.77
BFI-10 Neuroticism/10	5.97 ± 1.85
BFI-10 Conscientiousness/10	7.83 ± 1.44

BFI, Big-Five-Inventory; SD, standard deviation; VLMT, Verbal Learning and Memory Test.

**Table 2:** Self-reported subjective cognitive complaints.

Questionnaire	Mean ± SD
Everyday Cognition (ECog)	
Total score*/156	52.03 ± 13.18
Memory/32	13.67 ± 4.49
Language/36	12.43 ± 4.05
Visuospatial perception/28	7.60 ± 1.89
Executive functions: Planning/20	5.63 ± 1.45
Executive functions: Organization/24	6.73 ± 1.70
Executive functions: Divided attention/16	5.97 ± 2.14
Memory Functioning (MFQ)	
Total score/441	283.77 ± 38.78
Retrospective functioning/35	17.57 ± 4.20
Frequency of forgetting/126	91.63 ± 15.97
Frequency of forgetting during reading/70	54.83 ± 10.80
Remembering past events/28	16.17 ± 4.43
Seriousness of forgetting/126	77.1 ± 23.31
Mnemonics usage/56	26.47 ± 6.52

For ECog, a greater value indicates more subjective cognitive complaints. For MFQ, a greater value indicates fewer subjective cognitive complaints. *Average response rating (/4): 1.35 ± 0.34.

### 3.2. Visual processing speed *C*, age, and objective cognitive performance

The mean and SD of visual processing speed *C* estimates and their correlations with age are shown in Table 3. Visual processing speed *C* did not significantly correlate with age (rho = −0.31, *p* = 0.099; Table 3) or with the overall cognitive performance *z*-score (*p* = 0.275; controlling for age: *p* = 0.670) in the current sample. The remaining visual attention parameters’ mean, SD, and correlations with age are listed in Table 3. Notably, higher VSTM storage capacity *K* (rho = 0.58, *p* = 0.001; controlling for age: rho = 0.54, *p* = 0.003) was associated with better overall objective performance.

**Table 3:** TVA parameters and their correlation with age.

	Visual processing speed *C* [letters/s]^a^ (*p*-value)	VSTM storage capacity *K* [max. # of letters] (*p*-value)	Top-down control *α* [distractors relative to targets]^b^ (*p*-value)	Spatial weighting *w_lat_* [left relative to right]^b^ (*p*-value)
Mean ± SD	27.20 ± 10.02	2.70 ± 0.60	0.47 ± 0.28	0.48 ± 0.10
Correlation with age	−0.31 (0.099)	−0.25 (0.176)	0.18 (0.341)	0.13 (0.496)

^a^Sample’s visual perceptual threshold, t_0_, parameter = 30.46 ± 16.40 ms; ^b^*n* = 29 due to missing data of one observation.

### 3.3. Association between visual processing speed *C* and subjective cognitive complaints

**Figure 3:**
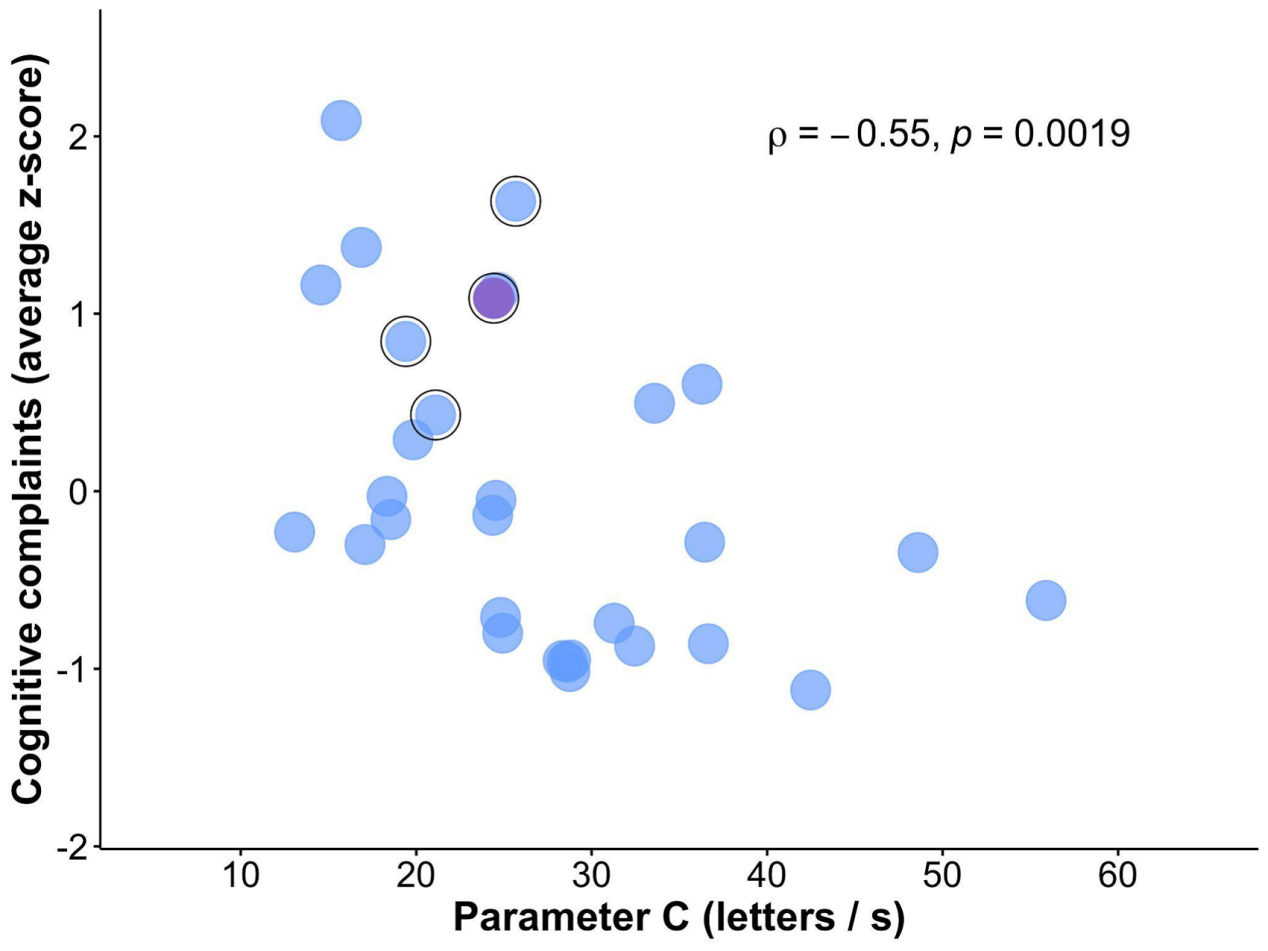
Scatterplot of the association between subjective cognitive complaints and visual processing speed *C*. Relationship between visual processing speed *C* and subjective cognitive complaints (i.e., *z*-score averaged across ECog and MFQ total scores). Outlined in black are participants that reported being concerned about their memory in the demographic questionnaire. Marked in violet is the participant who visited a doctor due to those memory concerns.

As hypothesized, visual processing speed *C* negatively correlated with subjective cognitive complaints (rho = −0.55, *p* = 0.002; Figure 3), indicating that *lower* visual processing speed *C* was associated with *greater* subjective cognitive complaints. The correlation remained significant when controlling for age, depressive symptoms, and education (rho = −0.48, *p* = 0.010; Table 4). The association with subjective cognitive complaints found for *C* was not observed for VSTM capacity *K*, top-down control *α*, or spatial weighting *w_lat_* (all *p*-values >0.533; controlling for age, education, and depressive symptoms: all *p*-values >0.158; Table 4). The multiple linear regression model showed that the association with subjective cognitive complaints was specific for visual processing speed *C*, compared to the other TVA parameters (*b* = −0.04, standard error = 0.02, *p* = 0.027).

**Table 4:** Correlation of TVA parameters with subjective cognitive complaints with and without controlling for age, education, and depressive symptoms.

	Visual processing speed *C* (*p*-value)	VSTM storage capacity *K* (*p*-value)	Top-down control *α* (*p*-value)	Spatial weighting *w_lat_* (*p*-value)
Overall subjective cognitive complaints [*z*-score]	−0.55 (0.002^*^)	−0.12 (0.533)	−0.10 (0.596)	−0.008 (0.968)
Overall subjective cognitive complaints [*z*-score] controlling for covariates	−0.48 (0.010^*^)	0.04 (0.844)	−0.29 (0.159)	0.02 (0.913)

^*^Significant at the Bonferroni-corrected *p*-value = 0.0125.

## 4. Discussion

In this study, we investigated the relationship between visual processing speed, measured by a psychophysical whole-report task and estimated with TVA-based fitting, and subjective cognitive complaints, measured by two structured questionnaires. We found that reduced visual processing speed is indeed associated with greater subjective cognitive complaints. Control analyses revealed that this association was not explained by age, education, or depressive symptoms. Further control analyses also revealed that no other visual attention parameter, such as visual short-term memory or top-down control, correlated with subjective cognitive complaints. Thus, our results indicate an objective slowness in visual processing, that is measurable with a sensitive measure for subtle changes in this basic parameter, to be a relevant neurocognitive correlate of subjective cognitive complaints. Our findings imply, first, that reductions in visual processing speed could interfere with older adults’ everyday tasks. Second, they imply that subjective cognitive complaints might foreshadow broader cognitive impairments. Overall, our study underscores the importance of examining the neurocognitive correlates of the subjective perception of cognitive function in healthy older adults.

Our study relies on a theory-grounded assessment of separable visual attention parameters as well as on a comprehensive assessment of objective and subjective cognitive functions. This strength allowed us to more precisely determine that the association with subjective cognitive complaints was specific for visual processing speed among other well-defined visual attention parameters in cognitively normal, community-dwelling older adults. Alzheimer’s disease dementia has been associated with a staged decline in visual processing speed (28), which is observed in patients at high risk for dementia (15, 25). In our study, the visual processing speed parameter showed sensitivity to subjective cognitive complaints in healthy older adults who score normally on standard neuropsychological tests. Neuroticism and conscientiousness personality factors, and especially depressive symptoms, although potentially relevant in this context (12, 26, 37), did not correlate with subjective cognitive complaints. Moreover, neither age nor depressive symptoms explained the link between visual processing speed and subjective cognitive complaints. Therefore, our results suggest that those with greater subjective cognitive complaints may be at risk for future broader cognitive impairments. Previously, subjective complaints of problems during wayfinding in familiar streets in healthy older adults have been shown to be closely associated with the odds of cognitive decline (29). On the other hand, recent evidence has shown that visual processing speed can be modulated through phasic alertness manipulations [e.g., (17)] or targeted cognitive interventions [e.g., (22)] in healthy older adults. In this context, our current results set the ground for future longitudinal studies aimed at determining whether individual changes in the visual processing speed parameter also reflect changes in subjective cognitive complaints and/or incipient cognitive impairments in older age.

Two additional methodological strengths of our study are worth mentioning. First, the current sample comprised healthy older adults who were not selected based on the presence of subjective cognitive decline (i.e., subjective cognitive complaints *and* concerns about them), thus allowing us to separate the subjective complaints as such from help-seeking behavior. In this regard, our sample’s reports of memory worsening without concerns, memory worsening with concerns, and medical help-seeking due to those concerns approximate those reported in population-based studies (e.g., ~ 45, 9, and 2%, respectively) (18). Second, while we used a task paradigm that included both cued and uncued trials to align with recent studies on mild cognitive impairment and visual processing speed (15), we focused on the uncued trials only for the present study. Supplementary control analyses nevertheless revealed a significant association between the cued visual processing speed parameter and subjective cognitive complaints. This finding suggests that the association between visual processing speed *C* and subjective cognitive complaints was not due to using only part of the trials or a specific experimental condition.

Although at an uncorrected level, greater subjective cognitive complaints correlated with worse episodic verbal memory performance (Supplementary material), in line with previous results based on samples from memory clinics (8, 11) population-based studies (4), and with results based on meta-analyses (24). This observation would also support the suggestion that healthy older adults’ subjective cognitive complaints reflect, at least partly, their actual memory performance (4). Overall cognitive performance correlated with VSTM storage capacity but not with visual processing speed. This is in agreement with the proposed stability across experimental conditions of this visual attention parameter (10) and with the fact that the neuropsychological measures are more related to memory than attention.

Our study has some limitations. First, as our sample is relatively small, our main results may be inconclusive until they are replicated in a larger sample. Second, we cannot determine whether a similar association between visual processing speed and subjective cognitive complaints also holds in participants recruited from memory clinics. On the other hand, clearer-cut relationships may be possible when testing these samples. Despite these limitations, our method and findings are informative for future, larger-scale multimodal studies on the trajectories of subjective cognitive complaints.

In conclusion, lower visual processing speed is associated with greater subjective cognitive complaints in community-dwelling healthy older adults. This association was still found when controlling for, and thus, was not explained by age, education, or depressive symptoms. Overall objective cognitive performance in standard neuropsychological tests was not related to visual processing speed. Taken together, our findings indicate that subjective cognitive complaints reflect a reduction in a basic neurocognitive mechanism, namely, visual processing speed. Importantly, these findings suggest that the combined assessment of subjective cognitive complaints and visual processing speed has the potential to identify individuals at risk for cognitive impairment (e.g., for research studies) early on, before the standard tests show any abnormal results. Ultimately, longitudinal measurements would allow extracting the predictive potential of this visual attention parameter.

## Data availability statement

The datasets and analysis scripts presented in this study can be found in the OSF repository and can be found at: https://osf.io/md6r9/

## Ethics statement

The study was approved by the Ethics Committee of the Faculty of Psychology and Education of LMU Munich (Munich, Germany) and was conducted in accordance with the Declaration of Helsinki. The participants provided their written informed consent to participate in this study.

## Author contributions

DM-P: data curation, formal analysis, investigation, software, visualization, writing—original draft, and writing—review and editing. KF: resources, supervision, and writing—review and editing. NR: investigation and writing—review and editing. MH: methodology, resources, software, and writing—review and editing. CR-M: investigation and writing—review and editing. AR-R: conceptualization, data curation, formal analysis, funding acquisition, project administration, visualization, writing—original draft, and writing—review and editing. All authors contributed to the article and approved the submitted version.

## Funding

This work was supported by the European Union’s Framework Programme for Research and Innovation Horizon 2020 (2014–2020) [Marie Skłodowska-Curie Grant Agreement No. 754388 (LMUResearchFellows)] and LMUexcellent [funded by the Federal Ministry of Education and Research (BMBF), the Free State of Bavaria under the Excellence Strategy of the German Federal Government and the Länder], and the LMU Neuro-Cognitive Psychology master’s program. Open Access funding enabled and organized by the LMU Research Fellowship Cooperation Funds.

## Conflict of interest

The authors declare that the research was conducted in the absence of any commercial or financial relationships that could be construed as a potential conflict of interest.

## Publisher’s note

All claims expressed in this article are solely those of the authors and do not necessarily represent those of their affiliated organizations, or those of the publisher, the editors and the reviewers. Any product that may be evaluated in this article, or claim that may be made by its manufacturer, is not guaranteed or endorsed by the publisher.

## Footnotes

^1^A value of 100 indicates complete right-handedness; −100, complete left-handedness; and 0, ambidexterity.

^2^This paradigm was used to facilitate comparison with previous studies examining tonic and phasic alertness in aging. However, in the present study, we do not aim for the assessment of these aspects. Therefore, here we focus only on *C* from the trials without auditory cues to rule out the cuing influences on *C* [as shown in, e.g., (15)].

^3^We used these two different frequencies to ensure an alerting effect of the tone cue by avoiding the habituation to one particular tone.
