# Supplementary material for: Lower visual processing speed relates to greater subjective cognitive complaints in community-dwelling healthy older adults
Source: Front Psychiatry. 2023 Mar 21;14:1063151. doi: 10.3389/fpsyt.2023.1063151 (PMC10072281; doi:10.3389/fpsyt.2023.1063151)
Supplement: Supplementary file 1 [file Data_Sheet_1.docx]

Supplementary Material for:

Lower Visual Processing Speed Relates to Greater Subjective Cognitive Complaints in Community-Dwelling Healthy Older Adults

**Daniela Marrero-Polegre, Kathrin Finke, Naomi Roaschio, Marleen Haupt, Cristian Reyes-Moreno, Adriana L. Ruiz-Rizzo**

1. **Supplementary Methods**
   1. **Visual processing speed measurement**

To calibrate the seven exposure durations (ED) in the whole-report task, a practice task was conducted. In this task, four blocks of 12 trials each were presented to the participants. In each block, the 12 trials were grouped into 4 triplets of trials. Each triplet, in turn, consisted of two calibration trials (masked) and one ‘easy’ trial (i.e., it could be a 250-ms masked trial or a 200-ms unmasked trial) to keep the participant engaged in the task. The initial ED was 100 ms and it was increased or decreased for the next calibration triplet if the participant, respectively, did not correctly report at least one letter correctly in the current one. The final adjusted ED was the lowest ED reached in the practice task. Four additional higher ED values were then obtained from a set of possible EDs predefined in the task, based on the individually adjusted ED.

1. **Supplementary Results**
   1. **Objective cognitive performance**

Among all measures, only a lower VLMT total score (rho = -0.44, *p* = 0.016; controlling for age: rho = -0.41, *p* = 0.029) and lower VLMT recognition (rho = -0.44, *p* = 0.014; controlling for age: rho = -0.40, *p* = 0.030) correlated with greater subjective cognitive complaints. However, the results were not significant after the Bonferroni correction for multiple testing. None of the other neuropsychological tests or depression and personality factor scores correlated with subjective cognitive complaints (all *p*-values > 0.074; controlling for age: all *p*-values > 0.097; Table S1). The overall cognitive performance *z*-score was significantly correlated with subjective cognitive complaints (rho = -0.42, *p* = 0.021), although age seemed to explain this correlation (rho controlling for age = -0.34, *p* = 0.073). GDS scores were below the clinical cutoff (i.e., 5) across participants and were not correlated with subjective cognitive complaints (rho = 0.33, *p* = 0.074; controlling for age: rho = 0.25, *p* = 0.197).

- 1. **Control analyses**

We examined whether the correlation with subjective cognitive complaints still held when (a) the visual processing speed *C* derived from the auditorily cued trials and (b) the parameter *C* averaged across both cued and uncued were used. Cued visual processing speed *C* (i.e., 27.52 ± 8.63 letters/s) significantly correlated with subjective cognitive complaints (rho = -0.41, *p* = 0.026) but this association appeared to be explained by age (controlling for age: rho = -0.37, *p* = 0.053; *n* = 29, due to a negative cued perceptual threshold of one participant). Similarly, the average of both (cued and uncued) *C* parameter estimates (i.e., 27.36 ± 8.21 letters/s) also correlated significantly with subjective cognitive complaints (rho = -0.54, *p* = 0.003; controlling for age: rho = -0.49, *p* = 0.008; *n* = 29).

1. **Supplementary Tables**

**Table S1.** Neuropsychological performance of the sample (*n* = 30) and its correlation with subjective cognitive complaints.

| **Test** | **Correlation with subjective cognitive complaints**  **z-score** *(p-*value*)* | **Correlation with subjective cognitive complaints**  **z-score controlling for age** *(p-*value*)* |
| --- | --- | --- |
| ***Overall Cognition*** |  |  |
| *z*-score across all tests | -0.42 (0.021) | -0.34 (0.073) |
| Crystallized Intelligence (IQ) | -0.08 (0.682) | -0.09 (0.632) |
| Addenbrooke’s Cognitive Examination III /100 | -0.13 (0.498) | 0.01 (0.968) |
| ***Memory*** | | |
| VLMT total learning score /75 | **-0.44 (0.016*^ns^*)** | **-0.41 (0.029*^ns^*)** |
| VLMT delayed recall /15 | -0.31 (0.090) | -0.31 (0.097) |
| VLMT recognition /15 | **-0.44 (0.014*^ns^*)** | **-0.40 (0.030*^ns^*)** |
| Rey-Osterrieth Complex Figure (delayed) /36 | -0.16 (0.387) | -0.03 (0.880) |
| ***Attention*** |  |  |
| Trail Making Test A (time in s) | -0.13 (0.497) | -0.22 (0.250) |
| ***Visuoconstruction*** | | |
| Rey-Osterrieth Complex Figure (copy) /36 | -0.20 (0.301) | -0.14 (0.454) |
| ***Executive Function*** | | |
| Trail Making Test B (time in s) | -0.11 (0.568) | -0.05 (0.809) |
| Stroop Test Interference (time in s) | 0.12 (0.544) | 0.06 (0.747) |
| ***Behavioral questionnaires*** | | |
| Geriatric Depression Scale /15 | 0.33 (0.074) | 0.25 (0.197) |
| BFI-10 Neuroticism /10 | 0.02 (0.929) | -0.003 (0.99) |
| BFI-10 Conscientiousness /10 | -0.20 (0.277) | -0.11 (0.566) |

Note. Significant correlations are in boldface; *ns* = Non-significant at the Bonferroni-corrected *p*-value = 0.004. BFI: Big-Five-Inventory; SD: standard deviation; VLMT: Verbal Learning and Memory Test
